# Licit and illicit substance use patterns among university students in Germany using cluster analysis

**DOI:** 10.1186/s13011-017-0128-z

**Published:** 2017-10-23

**Authors:** Laura Schilling, Hajo Zeeb, Claudia Pischke, Stefanie Helmer, Andrea Schmidt-Pokrzywniak, Ralf Reintjes, Ulla Walter, Maria Girbig, Alexander Krämer, Andrea Icks, Sven Schneider

**Affiliations:** 1 Mannheim Institute for Public Health, Social and Preventive Medicine, Heidelberg University, Ludolf-Krehl-Str. 7-11, D-68167 Mannheim, Germany; 2 Leibniz-Institute for Prevention Research and Epidemiology –BIPS, Achterstr. 30, D-28359 Bremen, Germany; 3 Institute of Medical Epidemiology, Biostatistics and Informatics, Faculty of Medicine, Martin-Luther-University Halle, Ernst-Grube-Str. 40, D-06120 Halle, Germany; 4 Department Health Sciences, Faculty Life Sciences, Hamburg University of Applied Sciences, Lohbrügger Kirchstr. 65, D-21033 Hamburg, Germany; 5 Department of Epidemiology, Social Medicine and Health System Research, Hannover Medical School, Carl-Neuberg-Str. 1, D-30623 Hannover, Germany; 6 Faculty of Medicine Carl Gustav Carus, Institute and Policlinic for Occupational and Social Medicine, TU Dresden, Fetscherstr. 74, D-01307 Dresden, Germany; 7 Faculty of Health Sciences, Bielefeld University, Universitätsstr. 25, D-33615 Bielefeld, Germany; 8 Institute of Health Services Research and Health Economics, Centre for Health and Society, Faculty of Medicine, Heinrich Heine University, Moorenstr. 5, D-40225 Düsseldorf, Germany

**Keywords:** Alcohol, Tobacco, Cannabis, Hookah, University students, Cluster analysis

## Abstract

**Background:**

The use of multiple licit and illicit substances plays an important role in many university students’ lives. Previous research on multiple substance use patterns of university students, however, often fails to examine use of different illicit substances and/or hookah. Our objective was to complement and advance the current knowledge about common consumption patterns regarding illicit substances and hookah use in this group.

**Methods:**

Students from eight German universities completed an online survey as part of the INSIST study (‘INternet-based Social norms Intervention for the prevention of substance use among STudents’) regarding their consumption of alcohol, tobacco, hookah, cannabis and other illicit substances. Cluster analysis identified distinct consumption patterns of concurrent and non-concurrent substance use and multinomial logistic regressions described key sociodemographic factors associated with these clusters.

**Results:**

Six homogeneous groups were identified: ‘Alcohol Abstainers’ (10.8%), ‘Drinkers Only’ (48.2%), ‘Drinkers and Cigarette Smokers’ (14.6%), ‘Cannabis and Licit Substance Users’ (11.2%), ‘Hookah Users with Co-Use’ (9.8%) and ‘Illicit Substance Users with Co-Use’ (5.4%). Illicit substance use clustered with the consumption of alcohol, tobacco and cannabis. Hookah use was regularly associated with alcohol consumption, less commonly associated with tobacco or cannabis use and very rarely associated with use of other illicit substances. Individuals consuming licit and illicit substances or hookah were mostly male and lived together with other students. Characteristics such as the number of years an individual had spent studying at a university, subject of study, immigrant background and religious affiliation were less commonly associated with cluster membership.

**Conclusions:**

Although we found substance use patterns in our sample largely similar to previous reports, we identified an important subgroup of individuals using both illicit and licit substances. These individuals may benefit especially from targeted interventions that focus on modifying addictive behavior patterns.

**Trial registration:**

DRKS00007635. Registered 17 December 2014 (retrospectively registered).

## Background

Recent trends such as the spread of hookah use, the emergence of new forms of synthetic substances and a growing discussion surrounding legalization of cannabis illustrate the immediacy and relevance of changing licit and illicit substance consumption patterns in today’s society [1–3]. Consumption of every type of licit and illicit substance is an important health risk factor [4, 5]. Especially in young adulthood, substance use can lead to injuries, suicide and ultimately lead to death [6–8].

Early adulthood is an important period in the lifespan as behaviors such as substance use are often initiated and established during this time. This is particularly true for young adults attending university as the transition to this new setting is associated with a change in living environment and social networks [9, 10]. Overall alcohol consumption and binge drinking in university students are, for example, higher than in young adults who do not attend university [11–13]. The prevalence of hookah use has also increased among university students and now exceeds cigarette use in some countries [3, 14]. Use of illicit substances is also higher in this group [10, 15–17]. According to one report of 3307 students at German universities, for example, the lifetime prevalence of cannabis use was 40% [18], while other work conducted in the German population suggests lower lifetime prevalence’s of 23.0% and 30.8% in the age groups 18–20 and 21–24 years, respectively [19].

Traditionally, many epidemiological studies focus on individual health-related behaviors such as the consumption of a single licit or illicit substance [20, 21]. However, recent research shows that students tend to consume more than one substance on a regular basis [18]. For example, students who smoke cigarettes or take illicit substances often drink more alcohol [18, 22, 23]. Use of multiple licit and illicit substances by the same individual is associated with social (e.g., relationship problems), psychological (e.g., aggressiveness) or health risks (e.g., risky sexual behavior) [24–29]. Co-use of tobacco and cannabis is associated with higher risk of depression and externalizing personality risk factors (e.g., delinquency, aggression and hostility) [30].

Previous work suggests the value of assessing clusters of multiple substance use behaviors as a way to identify important patterns that can be used to target future interventions. A majority of studies, however, group substance use (e.g., alcohol, tobacco and/or cannabis) with other health-related behaviors (e.g., nutrition, sports) or group only substances like alcohol, tobacco, hookah and cannabis, but no other illicit substances [21, 31–34]. This makes it difficult to identify specific patterns of licit and illicit substance use. Our understanding of this topic is currently limited to a single study conducted in the U.S. in which substance use patterns in a cohort of first year university students were examined [35]. The comprehensiveness of this study may be limited, however, as it did not assess hookah, an increasingly common substance used by university students with abuse potential and associations with other types of problematic substance use [2, 35].

The objective of this paper, therefore, is to advance current knowledge about common consumption patterns of licit and illicit substances, including hookah among university students. A secondary aim is to identify sociodemographic and other characteristics associated with these consumption patterns as a first step to inform the development of targeted prevention and intervention programs.

## Methods

### Participants and procedure

Our analysis used data from the baseline survey of the INSIST study (‘INternet-based Social norms Intervention for the prevention of substance use among STudents’). The goals of INSIST were to investigate whether a social norm intervention lead to a significant reduction of licit and illicit substance use in university students studying in Germany. INSIST participants were recruited from eight universities throughout Germany using a multi-channel approach (e.g., via students’ directories, social media and through student bodies) to minimize selection bias [15]. Eligibility for participation was based on registration at one of the university sites, age > 17 and ability to complete a questionnaire in German. The INSIST survey, based on previously validated instruments and items, underwent expert review, was discussed in focus groups, and was pretested with university students. Intervention effectiveness was assessed by online survey using a pre-post test design. Further details on study procedure and questionnaire development can be found elsewhere [15]. The Ethics Committee of the Hannover Medical School provided initial study approval and this was forwarded for review and approval by the Ethics Committees at the other universities sites.

A total of 4387 subjects provided complete information in the baseline survey on consumption of licit and illicit substances and were therefore included in the current analysis.

### Measurement and operationalization

#### Substance use

Our survey evaluated both concurrent and non-concurrent usage behavior. Consumption frequency was assessed using the item: ‘How often have you consumed the following substances in the last two months?’. Response categories included ‘never in my life’, ‘have consumed, but not in the last two months’, ‘once in the last two months’, ‘twice in the last two months’, ‘once every two weeks in the last two months’, ‘once per week in the last two months’, ‘twice per week in the last two months’, ‘three times per week in the last two months’, ‘four times per week in the last two months’ or ‘every or nearly every day in the last two months’. As we were more interested in usage patterns rather than frequency, we collapsed ordinal response categories into a binary indicator according to the gold standard of licit and illicit substance use in younger populations [34, 36]: 0 = ‘no consumption of alcohol in the last two months’ and 1 = ‘consumption of alcohol in the last two months’. Substance assessment categories included alcoholic drinks (e.g., beer, wine, liquor), tobacco (e.g., cigarettes, cigars), cannabis (e.g., marijuana, ‘weed’) and hookah (e.g., shisha – this category excluded cannabis in a ‘bong’).

Use of other illicit substances, including synthetic cannabis (e.g., spice), cocaine (e.g., coke, crack, freebase), ecstasy, hallucinogens (e.g., LSD, magic mushrooms, trips), inhalants (e.g. solvent, glue, petrol), and other amphetamines/stimulants (e.g., speed, pep, crystal) was elicited by a similar item referencing use of each respective substance. The response for other illicit substances was measured on a scale of 1 to 5 (‘never in my life’, ‘have consumed, but not in the last two months’, ‘one to three times in the last two months’, ‘weekly or more frequently in the last two months’ or ‘every or nearly every day in the last two months’. In contrast to cannabis use, the 2-month prevalence of each of the substances above was <3%. We therefore created a binary indicator for use of any other illicit substance (0 = ‘no consumption of other illicit substances in the last two months’ and 1 = ‘consumption of other illicit substances in the last two months’).

#### Sociodemographic characteristics

We assessed several sociodemographic characteristics including gender, age, living situation, and any religious affiliation in line with previous work [16, 22, 34]. We included a binary indicator for any religious affiliation instead of an indicator for a specific religion, because the number of respondents who reported having a religion other than Christianity was very low (<1% for Muslim; Jewish; Hindu; Buddhist). Although a specific question on ethnicity was not part of the INSIST survey, we included an assessment of acculturation using a modified version of a previously published definition of immigrant status [37]: A participant was considered to be an immigrant if both parents were born in another country, if the individual had not lived in Germany since birth and at least one parent had been born in another country, or if an individual reported speaking a language other than German at home.

We also included indicators for the subject of study and the number of years a student had been studying at a university in our analysis. Previous work indicates, for example, that students from sporting or medical faculties have a higher risk of heavy and problematic drinking behavior, while another study showed that the lifetime prevalence of cannabis use is more frequent among arts and social science students [38–42]. Research similarly suggests associations between years of university and substance use [34, 42].

### Statistical analyses

After an initial descriptive analysis, a cluster analysis was employed to identify patterns of substance use among university students. Cluster analysis is a multivariate technique for identifying homogeneous subgroups within heterogeneous samples. It was applied in the current study to identify common consumption patterns and to inform the development of prevention and interventions programs, especially those that might target subgroups with high-risk or more problematic consumption patterns behaviors. Although this complex method is widespread in sociology and commercial market research, it is not frequently used in epidemiological and addiction studies [43, 44].

Due to the high number of cases and the binary structure of the variables, we chose a two-step cluster analysis approach [45]. The analysis was initially performed with binary indicators for the five categories of substance use over the previous two months: Consumption of alcohol, tobacco, hookah, cannabis or other illicit substances. Because the exact number of identifiable clusters was not known a priori, a two-step algorithm’s automatic clustering function was used, in which the ratio of distances between the clusters was calculated using Schwarz’s Bayesian Criterion (BIC). A large ratio of distances is usually associated with the optimal number of clusters [45]. In a post hoc analysis, we used two-sided chi square test and a multinomial logistic regression with stepwise selection to assess associations between cluster membership and participants’ sociodemographic characteristics. The pre-defined level of significance was *p* < 0.05. All analyses were conducted using SPSS 22 Statistics (IBM Corporation, Somers, NY 10589, USA).

## Results

The sociodemographic characteristics of the participants are presented in Table 1. Mean age of the sample was 23.7 years (SD 4.00).

**Table 1:** Sociodemographic characteristics of the university students

Sociodemographic characteristics	n = 4387 (%)
Gender	
Male	41.5%
Female	58.5%
Age	
< 23 years	42.2%
23 to 27 years	45.4%
> 27 years	12.4%
Immigrant background	
Immigrant background	8.7%
No immigrant background	91.3%
Religious affiliation	
Religious affiliation	51.0%
No religious affiliation	49.0%
Living situation	
With other students	41.3%
Alone or with partner	45.5%
With parents	13.1%
Years of studying at a university	
1st year undergraduate	20.7%
2nd year undergraduate	19.3%
3rd year undergraduate	16.6%
4th year undergraduate	9.9%
5th year undergraduate	11.9%
Graduate	21.6%
Subject of study	
Humanities	6.6%
Health Care, Medicine, Sport	16.9%
Natural Science	19.6%
Economics, Law	10.7%
Engineering	14.1%
Linguistics, Cultural Sciences	10.8%
Social, Educational Science	21.3%

percentages are based on valid cases

The use prevalence of licit substances like alcohol and tobacco was relatively high in the study sample, while use of cannabis was less common (Table 2). Although less frequent, use of other illicit substances over the previous two months was reported for amphetamines and/or stimulants (2.9%), ecstasy (2.7%), hallucinogens (1.5%), cocaine (1.1%), synthetic cannabis (1.0%) and inhalants (0.3%).

**Table 2:** Consumption behavior of the university students by gender

Consumption of substances	Female *n* = 2566 (%)	Male *n* = 1817 (%)	Total *n* = 4387 (%)
Consumption of alcohol in the last two months			
Never	2.9%	2.9%	2.9%
Not in the last two months	8.7%	6.8%	7.9%
At most twice a week	76.3%	64.9%	71.6%
Three times a week or more often	12.0%	25.3%	17.5%
Consumption of tobacco products in the last two months			
Never	42.0%	30.8%	37.3%
Not in the last two months	30.1%	31.2%	30.5%
At most twice a week	13.8%	20.6%	16.6%
Three times a week or more often	14.1%	17.4%	15.5%
Consumption of cannabis in the last two months			
Never	57.4%	37.9%	49.3%
Not in the last two months	29.0%	33.5%	30.9%
At most twice a week	12.0%	22.6%	16.4%
Three times a week or more often	1.6%	6.1%	3.5%
Consumption of hookah in the last two months			
Never/not in the last two months	91.7%	85.4%	89.1%
At least once in the last two months	8.3%	14.6%	10.9%
Consumption of other illicit substances in the last two months			
Never/not in the last two months	95.9%	92.4%	94.5%
At least once in the last two months	4.1%	7.6%	5.5%

percentages are based on valid cases

### Typical licit and illicit substance user groups

**Fig. 1:**
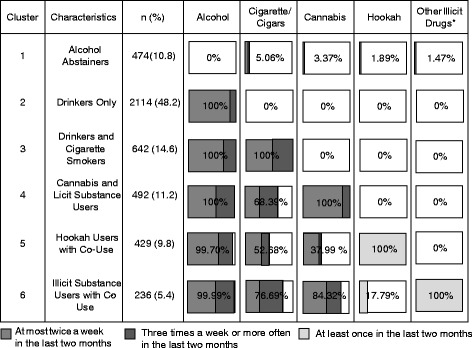
2-month-prevalence of substance use within the clusters (*n* = 4387); percentages are based on valid cases; note: *synthetic cannabis, cocaine, ecstasy, hallucinogens, inhalants, other amphetamines and/or stimulants

In addition to a less useful two cluster solution (cluster 1: ‘Global Abstainers or Drinkers’; Cluster 2: ‘Multiple Substance Users’; BIC: 2.496), the BIC suggested a well-differentiated 6-cluster solution (BIC: 1.804). This 6-cluster solution allowed for the intuitive identification of patterns within the specific illicit substance and hookah use groups. These clusters were labeled according to the predominant substance(s) use (Fig. 1).

The largest subgroup in the sample (cluster 2) drank alcoholic beverages, usually at a moderate level, but did not report consuming other substances. Most other clusters were characterized by the consumption of substances. One cluster incorporated hookah use combined predominantly with alcohol consumption at a moderate level and less often with tobacco and cannabis use (cluster 5). Hookah users were infrequently represented in groups using other illicit substances. Those reporting use of illicit substances other than cannabis (cluster 6) regularly drank alcohol and smoked tobacco, occasionally consumed cannabis, but infrequently smoked hookah.

### Socio-demographic characteristics of the cluster members

**Fig. 2:**
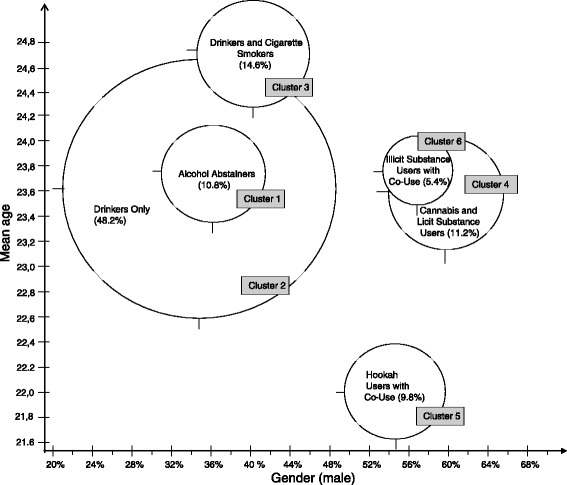
Cluster description on the basis of gender and age (n = 4387)

We observed significant associations between cluster membership and all sociodemographic and student-related variables (*p* < 0.001). The gender and age-specific structure of the individual clusters is illustrated in a two-dimensional matrix (Fig. 2). Here, we noted that the gender of cluster members reporting consumption of licit and illicit substances (clusters 4, 5, 6) was primarily male. In contrast, those in clusters 1 and 2 (‘Alcohol Abstainers’ and ‘Drinkers only’) were predominately females. ‘Hookah Users with Co-Use’ (cluster 5) were generally younger compared to members of the other clusters (Fig. 2).

Using members of cluster 1 (‘Alcohol Abstainers’) as the reference group, we used multinomial regression to explore further the association of individual sociodemographic characteristics with cluster membership. The odds of male gender was highest in groups in which illicit substances use predominated (clusters 4, 5, 6) compared to the reference group (cluster 1). For example, ‘Cannabis and Licit Substance Users’ were 2.6-times more likely to be male (95%-CI 1.99, 3.56; *p* < 0.001). ‘Hookah Users with Co-Use’ was also strongly associated with the youngest age group (<23 years) with an odds ratio of 7.07 (95%-CI 3.34; 14.96; p < 0.001).

Those not from an immigrant background and those with any religious affiliation were commonly represented in cluster 2 (‘Drinkers Only’). Students from immigrant backgrounds were 0.5 times less likely (95%-CI 0.37; 0.70; p < 0.001) and those reporting any religious affiliation were 1.4 times more likely to be in this cluster (95%-CI 1.14; 1.74; p < 0.001).

A student‘s living situation was also associated with cluster membership: those living with other students had a higher risk of being in a multiple substance use cluster: The strongest associations were observed in the group in which illicit substance use predominated (clusters 4 and 6): those living together with other students had a 5.3 higher probability of being in the group of ‘Cannabis and Licit Substance User’, compared to those in the reference group. In terms of a student’s subject of study, students in natural science were relatively less likely to be members of clusters 3 or 4 (Table 3).

**Table 3:** Results of the multinomial logistic regression for sociodemographic characteristics (*n* = 4238)

	n (%) 4238 (100%)	Cluster 2: ‘Drinkers Only’ (*n* = 2053)	Cluster 3: ‘Drinkers and Cigarette Smokers’ (*n* = 621)	Cluster 4: ‘Cannabis and Licit Substance Users’ (*n* = 469)	Cluster 5: ‘Hookah Users with Co-Use’ (*n* = 418)	Cluster 6: ‘Illicit Substance Use with Co-Use’ (*n* = 226)
Gender						
Male	1753 (41.4)	0.91 (0.72–1.15)	1.28 (0.98–1.68)	2.66 (1.99–3.56)***	2.05 (1.52–2.76)***	2.09 (1.47–2.99)***
Female	2485 (58.6)	ref	ref	ref	ref	ref
Age						
< 23 years	1792 (42.3)	1.29 (0.89–1.87)	0.46 (0.30–0.70)**	1.06 (0.64–1.76)	7.07 (3.34–14.96)***	0.71 (0.39–1.29)
23 to 27 years	1933 (45.6)	1.49 (1.08–2.04)*	1.00 (0.70–1.43)	1.52 (0.98–2.35)	4.96 (2.45–10.05)***	0.97 (0.58–1.64)
> 27 years	513 (12.1)	ref	ref	ref	ref	ref
Immigrant background						
Immigrant background	368 (8.7)	0.50 (0.37–0.70)***	0.59 (0.39–0.88)*	0.83 (0.54–1.27)	0.67 (0.42–1.05)	1.01 (0.61–1.68)
No immigrant background	3870 (91.3)	ref	ref	ref	ref	ref
Religious affiliation						
Religious affiliation	2164 (51.1)	1.41 (1.14–1.74)***	0.98 (0.77–1.26)	0.85 (0.65–1.12)	1.12 (0.85–1.48)	0.71 (0.51–1.00)
No religious affiliation	2074 (48.9)	ref	ref	ref	ref	ref
Living situation						
With other students	1759 (41.5)	2.28 (1.66–3.12)***	3.04 (2.03–4.57)***	5.32 (3.44–8.24)***	3.39 (2.25–5.11)***	4.88 (2.86–8.34)***
Alone or with partner	1929 (45.5)	1.37 (1.02–1.82)*	1.72 (1.18–2.53)*	1.29 (0.84–2.00)	1.18 (0.78–1.76)	1.30 (0.75–2.24)
With parents	550 (13.0)	ref	ref	ref	ref	ref
Years of university						
1st year undergraduate	868 (20.5)	0.67 (0.45–0.98)*	1.32 (0.84–2.07)	0.97(0.58–1.59)	1.05 (0.61–1.78)	1.43 (0.79–2.59)
2nd year undergraduate	825 (19.5)	0.84 (0.57–1.23)	1.24 (0.79–1.96)	0.99 (0.60–1.63)	1.02 (0.60–1.74)	1.22 (0.67–2.22)
3rd year undergraduate	704 (16.6)	0.67 (0.46–0.97)*	1.04 (0.68–1.60)	0.93 (0.58–1.48)	0.87 (0.51–1.46)	0.88 (0.49–1.58)
4th year undergraduate	419 (9.9)	0.64 (0.42–0.97)*	1.00 (0.62–1.62)	1.03 (0.61–1.72)	1.20 (0.68–2.10)	1.14 (0.61–2.14)
5th year or more undergraduate	507 (12.0)	0.62 (0.43–0.90)*	0.83 (0.54–1.28)	0.80 (0.49–1.30)	0.90 (0.52–1.57)	0.59 (0.31–1.13)
Graduate	915 (21.6)	ref	ref	ref	ref	ref
Subject of study						
Humanities	280 (6.6)	1.18 (0.71–1.95)	1.87 (1.08–3.24)*	1.23 (0.68–2.22)	1.33 (0.71–2.51)	1.36 (0.65–2.84)
Health Care, Medicine, Sport	725 (17.1)	1.38 (0.99–1.92)	0.89 (0.60–1.31)	0.61 (0.39–0.96)*	0.91 (0.57–1.44)	0.60 (0.33–1.10)
Natural Science	832 (19.6)	1.09 (0.80–1.50)	0.50 (0.33–0.74)**	0.56 (0.37–0.84)**	0.69 (0.45–1.06)	0.77 (0.46–1.28)
Economics or Law	456 (10.8)	1.32 (0.88–1.97)	1.31 (0.83–2.08)	0.63 (0.37–1.07)	1.34 (0.81–2.23)	1.10 (0.59–2.04)
Engineering	588 (13.9)	1.32 (0.89–1.94)	0.81 (0.51–1.28)	0.66 (0.41–1.06)	1.23 (0.76–1.99)	1.30 (0.75–2.26)
Linguistics, Cultural Sciences	452 (10.7)	1.12 (0.76–1.64)	1.08 (0.70–1.69)	0.99 (0.61–1.60)	0.95 (0.56–1.60)	1.15 (0.62–2.10)
Social or Educational Science	905 (21.4)	ref	ref	ref	ref	ref

cases with missing values were excluded from the analysis

percentages are based on valid cases

odds ratio (95%-confidence interval); *p < .05; **p < .01; ***p < .001

ref.: reference category (view Table 1)

reference category: Cluster 1 (‘Alcohol Abstainers’ *n* = 460)

model fit: Likelihood-Ratio Test: χ^2^ = 689.84; df = 90; McFadden Pseudo R^2^ = 0.054

## Discussion

The aim of our study was to identify common substance use patterns involving licit substances like alcohol, tobacco products and hookah and a wide range of illicit substances among students who are studying for a different length of time. Our analysis revealed six distinct clusters. Notably, three of these included either illicit substance and/or hookah use. A cluster identified by use of other illicit substances (including synthetic cannabis, cocaine, ecstasy, hallucinogens, inhalants, other amphetamines and/or stimulants) was also characterized by use of multiple substances.

A direct comparison of our results to those from other studies is difficult, even though we used definitions similar to those found in other research [34, 36]. The number of clusters vary to the study of Cho et al., they detected only three different groups for the consumption of alcohol, cannabis and other illicit substances [35]. Reasons for this could be cultural differences in the acceptance of using various substances, as well as different ways of measuring consumption [34, 35]. To better understand how illicit substance and hookah use cluster together, further work is needed in which the single and concurrent use of alcohol, tobacco, hookah, cannabis and other illicit substances are considered alongside different methods for detecting and measuring their use. Nevertheless the present study demonstrates that use of hookah and/or illicit drugs is also associated with the use of multiple other substances. Multiple substance use represents a potential indicator of addictive behavior, placing the user at greater health and social risks. Members of this subgroup may benefit especially from interventions that focus specifically on modifying these behavioral patterns [24–28, 46–48].

Consistent with previous research, we observed that illicit substance and hookah users are predominately male [22, 34, 49]. The current analysis, however, suggests that the association is strongest between male gender and the group of ‘Cannabis and Licit substance users’ compared to other clusters. Student living arrangements also appear important. Our findings suggest, for example, that students living with other students are more likely to use different substances with the highest odds ratios identified in members of the cluster ‘Cannabis and Licit Substance Users’. Previous work suggests that students living with other students have a higher risk of developing problematic consumption patterns and/or of engaging in other risky behaviors [32, 34, 50], while those living with their parents are at lower risk of using licit or illicit substances [22, 34]. This observation may be related to exposure to greater peer pressure to consume or increased access to licit and illicit substances through housemates that assist in their procurement [51–54]. In terms of age, younger undergraduate students appear more likely to use hookah. As with living arrangements, this finding may also relate to peer pressure and the perception among younger students that hookah use is more trendy [55, 56].

Identifying the sociodemographic and student-related characteristics associated with specific user subgroups can be helpful for developing targeted interventions. One example of such targeted interventions, which was also carried out in the INSIST study, are online-based ‘social norms interventions’. This form of intervention uses online feedback to inform people about their behavior compared with the behavior of the wider population [57]. As part of the INSIST study every participant (this includes especially students with most problematic substance use) got a personalized feedback via email. Past research suggests this kind of intervention can lead to a reduction of social ‘peer pressure’ and can possibly result in reduced substance use [58]. Given their focus on normative behavior, social norms interventions may be particularly valuable in addressing multiple substance use and individuals with specific characteristics (male, younger age groups, students living together with other students) [51].

Other features associated with cluster membership may be relevant for the implementation of intervention measures. Consistent with previous work, we note that students’ subject of study was associated with substance use behavior [38–41]. This finding may reflect personal characteristics related to the choice of a subject of study that are also related to substance use (e.g., female students with lower substance use study more frequently lectures) [41]. The odds ratios in the present study, as in previous research, are rather small [38]. Moreover we were unable to identify a specific subject of study at higher or lower risk for a pattern of multiple licit and illicit substance use due to limited power. Further exploration of this association using larger samples should prove helpful in determining its importance.

Students with an immigrant background were also less frequently found in clusters where only alcohol or/and tobacco were consumed. A Dutch study showed that students without a Dutch ethnic background consumed fewer substances than students with a Dutch ethnic background [31]. It is likely that different cultural attitudes, beliefs and practices account for some of these differences [59].

Finally associations between cluster membership and report of any religious affiliation could only be detected in cluster 2 ‘Drinkers Only’ and stand in contrast to previous work, which suggested a ‘protective’ association between religious practice and consumption of licit and illicit substances [60, 61]. Use of measures weakly reflecting constructs such as religiosity or acculturation [32].

### Strengths & limitations

To our knowledge, this is the first study worldwide to identify patterns of alcohol, tobacco products, hookah, cannabis or other illicit substance use in a sample of university students using the cluster analysis method, a novel and innovative application in the field of Public Health. Moreover, INSIST offers one of the largest datasets on substance use behaviors in students from multiple regions and educational institutions across Germany.

Our findings should be interpreted with several limitations in mind, however. Our sample was not representative of the general German student population: The proportion of males and median age in the study sample was 11% points and 0.5% points lower, respectively, than national figures [62, 63]. This may have resulted from differences in the method and recruitment strategy at each university, a higher willingness to participate of the female students and/or the language in which the questionnaire was administered. International students, who comprise between 7 and 19% of the student bodies at participating universities, were not eligible for participation if they could not also understand German. Second, we limited our analysis to cross-sectional data from the baseline survey of the INSIST study, providing a single snapshot in time of behaviors that may change significantly for individuals and across time. Third, our cross-sectional study design does not support causal inferences. For example, it is unclear whether a student was more likely to consume illicit substances because s/he lived with another student or that a student who reports consumption of illicit substances is more likely to live with other students. Since we employ measures of prevalence over an extended period, the cross-sectional design of our study may be less important in interpreting the value of our study. Fourth, we rely on self-reports of substance use, a sensitive subject with legal implications. In general, studies have shown that self-report of alcohol consumption are valid [64]. Social desirability, however, may have affected responses in other areas such as illicit substance use or for licit substances in which age of consumption is strictly regulated [65]. Fifth, our results may differ from previous work due to widely differing definitions and measurements strategies across studies [34, 66]. For example, the 2-month prevalence rate of tobacco smokers among students in our sample was 8% points lower than the 2-month prevalence in another study [67]. Finally we used binary indicators for licit and illicit substance use. While this approach is thought to represent the gold standard by some [36] and increases power to detect meaningful associations, a more graded approach may be useful in providing greater detail on specific patterns.

## Conclusion

Specific clusters of substance consumption appear to exist in which hookah use and/or illicit substance use predominates. Our findings further suggest that the members of these clusters appear particularly likely to use multiple other substances as well. As many health-related behaviors are established in early adulthood, greater attention must be focused on barriers and disincentives to adopting and sustaining behaviors that favor consumption of multiple licit and illicit substances [32]. Recognition of clusters of consumption behavior and their associated sociodemographic characteristics may be useful in informing the direction of future targeted prevention and intervention measures.

Further descriptive research on specific patterns of substance use and the correlates of these behaviors is needed. The pattern of multiple substance use in a significant proportion of university students, however, suggests a need for action. Unfortunately, few carefully evaluated, evidence-based interventions addressing multiple substance use behaviors currently exist. Future research should therefore focus on the development, evaluation and dissemination of interventions and best practices at the individual, group and environmental levels that seek to interrupt the health consequences of licit and illicit substance use behaviors in early adulthood.

## Abbreviations

BIC: Schwarz’s Bayesian Criterion
CI: Confidence Interval
INSIST: ‘INternet-based Social norms Intervention for the prevention of substance use among STudents’
OR: Odds Ratio
